# Prevalence of JC and BK Polyomavirus Infection in Patients with Chronic Kidney Disease in the State of Pará, Brazil

**DOI:** 10.3390/tropicalmed8010009

**Published:** 2022-12-23

**Authors:** Scheila do Socorro Vasconcelos Ávila da Costa, Jacqueline Cortinhas Monteiro, Ana Paula do Vale Viegas, Keyla Santos Guedes de Sá, Silvia Regina da Cruz, Sandra Souza Lima, Izaura Maria Vieira Cayres Vallinoto, Igor Brasil Costa, Antonio Carlos Rosário Vallinoto

**Affiliations:** 1 Laboratório de Virologia, Universidade Federal do Pará, Belém 66075-110, Brazil; 2 Programa de Pós-Graduação em Biologia de Agentes Infecciosos e Parasitários, Instituto de Ciências Biológicas, Universidade Federal do Pará, Belém 66075-110, Brazil; 3 Hospital Ophir Loyola, Belém 66063-240, Brazil; 4 Seção de Virologia, Instituto Evandro Chagas, Ananindeua 67030-000, Brazil

**Keywords:** Polyomaviruses, JC virus, BK virus, chronic kidney disease

## Abstract

The polyomaviruses that infect humans, JC virus (JCV) and BK virus (BKV), can establish persistent infections in the cells that make up the renal system, causing nephritis and BKV-associated nephropathy in up to 10% of renal transplant patients, and of these, 90% lose the graft and return for hemodialysis. This study aimed to determine the prevalence of polyomaviruses (PyV) in the population with chronic kidney disease (CKD), classified into three groups (conservative, dialysis, and transplanted) and a control group. Urine samples were collected from 290 individuals, including 202 patients with CKD and 88 from the control group. PyV screening was performed by PCR amplification of a fragment of the VP1 region, and the JCV and BKV species were distinguished through enzymatic digestion with the restriction endonuclease *BamHI* from the amplification of a TAg region. All amplification products were visualized on a 3% agarose gel. The prevalence of PyV infection was correlated with clinical-epidemiological variables using the chi-squared and Fisher’s exact tests. In the group with CKD, the prevalence of PyV was 30.2%, a higher rate being observed in conservative patients (36.66%; 22/60), followed by dialysis patients (30.48%; 25/82), and transplanted patients (20%; 12/60). In the control group, the prevalence was 46.59% (41/88). The differentiation between species revealed that JCV was present in 77.8% and BKV in 22.2% of the group with CKD. The prevalence of infection was higher in male patients (59.32%), whose most common pathology was systemic arterial hypertension (35.59%). In the group of transplanted patients, there was a statistically significant association between infection and the use of the immunosuppressant azathioprine (*p* = 0.015). The prevalence of PyV infection was higher in the control group than in the group with CKD, being predominant in males and in patients with systemic arterial hypertension.

## 1. Introduction

According to the International Committee of Viral Taxonomy, polyomaviruses belong to the family *Polyomaviridae,* comprising five genera and 80 species. The BK and JC viruses are included in the genus *Betapolyomavirus* and were renamed *human polyomavirus 1* and *human polyomavirus 2*, respectively [1]. They infect humans, monkeys, rats, birds, cattle, and rabbits. Each polyomavirus infects one type of host and rarely any other species. BK and JC viruses have the greatest clinical representativeness because they cause disease in humans [2,3].

More than 70% of individuals worldwide with serology tested for BKV have antibodies against this virus [4]. Primary infection occurs in childhood and is usually asymptomatic. However, under conditions of immunosuppression, the virus can leave the latency state and undergo reactivation. Kidney transplant recipients who receive intensive immunosuppressive drugs may develop BKV-associated nephropathy, with a risk of graft loss [5,6].

JCV is the causative agent of progressive multifocal leukoencephalopathy, which occurs in individuals with suppressed immune systems, especially in those with human immunodeficiency virus infection or acquired human immunodeficiency syndrome (AIDS) [7,8].

Primary BKV and JCV infections are typically subclinical or associated with mild respiratory symptoms [9,10], followed by dissemination to sites of persistent infection, especially renal and urinary tract cells [11,12]. Immunocompromised individuals are the main ones affected by diseases caused by polyomaviruses due to the reactivation of a latent subclinical state, resulting in an infection in the lytic phase, with viruria and viremia progressing to severe or even fatal diseases [13]. Their altered immunological conditions are caused by bone marrow, kidney, and other solid organ transplants or autoimmune diseases such as systemic lupus erythematosus and AIDS [14,15,16].

BKV-associated nephropathy (NBKV) is the most frequent disease related to this virus in renal transplant patients, manifesting as acute interstitial nephritis [17]. DNAuria and DNAemia, secondary to reactivation, are found in more than 80% of kidney transplant patients, and 10% of them progress to NBKV, which results in graft loss in approximately 90% of this subset over time [18,19,20,21]. The incidence of NBKV increased after the introduction of new and more potent immunosuppressive regimens [18,22,23], indicating the relationship between BKV reactivation and disruption of the immune system [13].

Given the importance to public health of the detection of polyomaviruses in immunosuppressed patients, which may cause disease when reactivated, this study aimed to quantify the prevalence of JC and BK viruses in patients with chronic kidney disease at different stages, according to the glomerular filtration rate (GFR), in order to plan future steps to prevent the reactivation of the virus after kidney transplantation.

## 2. Materials and Methods

### 2.1. Sampling

From June to November 2016, urine samples from 202 individuals with chronic kidney disease, treated at the Reference Units of the State of Pará, and 88 samples from the control group (no risk for the development of CKD) were analyzed. The patients involved in the study (*n* = 290) were divided into four groups: group 1 (G1) or conservative group, composed of 60 patients with CKD in the non-dialysis stages; group 2 (G2) or dialysis group, composed of 82 patients with CKD in stage 5; group 3 (G3) or transplanted group, composed of 60 kidney transplant patients; and group 4 (G4) or control group, composed of 88 patients with a low risk of developing CKD.

In the group of transplanted patients, the material was collected within 60 days after kidney transplantation. For the control group, individuals with a low risk of chronic kidney disease were screened according to the score applied in accordance with the CKD screening protocol of the Brazilian Society of Nephrology (SBN).

### 2.2. Inclusion and Exclusion Criteria

Patients aged 18 years or older were included in the study. The conservative, dialysis, and transplanted groups fit the definition of chronic kidney disease patients based on the National Kidney Foundation criteria [24]; in the dialysis group, patients with residual diuresis > 100 mL/day were included; in the control group, patients with a score ≤ 3 in the CKD screening protocol of SBN were included. All patients with unsatisfactory samples for analysis and/or who did not sign the informed consent form were excluded from the study.

### 2.3. Detection and Differentiation of the JC and BK Polyomaviruses

All urine samples were concentrated by centrifugation at 5000 rpm for 15 min, and the urinary sediment was washed three times with sterile saline solution (0.9%). The cell pellet was used for DNA extraction by the phenol–chloroform method. For detection of the polyomavirus, a 215-bp fragment of the VP1 gene was amplified using the JLP-15 (5′-ACAGTGTGGCCAGAATTCACTACC-3′) and JLP-16 (5′-TAAAGCCTCCCCCCCAACAGAAA-3′) primers as described by Agostini et al. [25].

For the differentiation of BKV from JCV, the samples that had an amplified VP1 region were subjected to PCR for the amplification of a 173-bp fragment of the TAg gene using the primers PEP-1 (5′-AGTCTTTAGGGTCTTCTACC-3′) and PEP-2 (5′-GGTGCCAACCTATGGAACAG-3′) as described by Arthur et al. [26]. Then, the amplicons were subjected to enzymatic digestion (TAg) using the *BamH* I restriction endonuclease (Invitrogen, CA, USA), which cleaved the JCV DNA into a 120-bp and a 53-bp fragment, while the BKV DNA was not cleaved. The products were visualized in a 3% agarose gel.

### 2.4. Ethics

The project was submitted to and approved (opinion no. 1486516) by the Research Ethics Committee of the Institute of Health Sciences of the Federal University of Pará, according to the norms established in resolution no. 466 of the National Health Council of 12 December 2012. The patients were invited to participate in the study and signed an informed consent form.

### 2.5. Statistical Analysis

The data obtained based on the questionnaires answered by the participants were added to the Epi-Info 7.2 database. Statistical analyses were performed using BioEstat 5.3 (Marimauá Institute, Manaus, Brazil) [27]. The qualitative data are presented as numbers and percentages. The categorical data were correlated using the chi-squared test and the *t* test, adopting a significance level of 5% (*p* < 0.05).

## 3. Results

The prevalence of infection was higher in male patients (59.32%), whose most common pathology was systemic arterial hypertension (35.59%). The mean age was 48.11 years. Patients in groups 1 and 2 had a slightly higher mean age (61.3 years and 55 years, respectively), while those in groups 3 and 4 had a mean age of 40.4 years and 37.1 years, respectively. Some 34.15% of patients with CKD had systemic arterial hypertension. The epidemiological characterization of the groups with CKD is shown in Table 1.

The global prevalence of polyomavirus was 35.17%, which was higher in the control group (46.6%) than in the groups with CKD (30.2%; *p* = 0.0072). The analysis of prevalence according to each group with CKD showed higher rates in the conservative group (36.7%; *p* = 0.0074) (Table 2).

Differentiation between JCV and BKV species was only possible in 24 samples from the conservative (G1) and control (G4) groups. In patients with CKD (group 1), JCV was detected in 77.8% and BKV in 22.2%, while in the control group, JCV was identified in 66.7% of cases and BKV in 33.3%.

Data regarding urea and creatinine levels were obtained for the conservative and dialysis groups, since they may influence PCR inhibitors. Among those with a positive result for polyomavirus, the median urea level in the conservative group was 54.5 mg/dL; however, in the dialysis group, it was 149 mg/dL. The median creatinine was 1.17 mg/dL in the conservative group and 7.16 mg/dL in the dialysis group.

The stratification of the GFR in the conservative group revealed that 41.7% (25%) of the patients had a GFR between 30 and 59 mL/min/1.73 m^2^, of whom 36% were infected with polyomavirus (Table 3).

In the transplanted group, the prevalence of polyomavirus was 20% (12/60). In the post-transplantation period, the immunosuppressive drugs of induction and maintenance were also analyzed, which may have influenced the degree of immunosuppression and thus the degree of reactivation of the polyomavirus, leading to viruria.

In the induction phase, it was observed that of the transplanted patients infected with polyomavirus, 28.6% used anti-thymocyte immunoglobulin (ATG) and 6.6% used IL-2 receptor inhibitors. In the maintenance phase, 40% of the patients used azathioprine, followed by mycophenolate (Table 4). A statistically significant association was observed only between infection and maintenance therapy, in which most infected patients used azathioprine (*p* = 0.015).

## 4. Discussion

Infections by polyomavirus are an important cause of graft loss in renal transplant patients. BK-associated nephropathy has become the most important complication observed in the posttransplant phase, with an estimated prevalence between 1 and 8% [28]. The reactivation of these viruses in renal transplant recipients remains a challenge for the medical team. It is extremely important to know the status of previous infection by these viruses in patients with CKD, especially those in the last stage, for clinical follow-up and for choosing the best therapeutic intervention. This study aimed to describe the prevalence rates of polyomavirus infection according to the stage of CKD and the GFR [24]. However, no association was observed between the rates found and the stage of the disease.

The prevalence rates of polyomavirus infection vary according to the population examined and the detection method used. In the present study, the prevalence of infection in patients with CKD was 30.2%, higher than the rates reported by Pires et al. [29] and [30], who reported prevalence rates of approximately 4% in urine samples from patients with CKD in the Brazilian cities of Belém (Pará state) and Acre, respectively. In contrast, in a study conducted in the metropolitan region of Belém involving the general population, Cayres-Vallinoto et al. detected the virus in 33% of the investigated samples. In the control group enrolled in the present study, the virus was detected in 46.6% [31].

Most earlier studies involving chronic kidney patients addressed the kidney transplant population, relating BKV to nephropathy, since this group has a high risk of graft loss (approximately 80%) when reactivation or new infection by BKV occurs. Thus, few studies report the prevalence of infection in the non-transplanted chronic kidney population. In Japan, Kaneko et al. [32] investigated patients with non-dialysis and non-transplanted CKD by PCR with amplification of the control region [33] and found 33.3% infection by BKV and JCV, corroborating the data found in the present study, where the prevalence of polyomaviruses in the conservative group was 36.7%. In patients treated at the Al-Karma hospital, Morocco, Ajeel et al. [34] observed that 16% of conservative patients and 40% of dialysis patients had BK polyomavirus infection, diverging from the data found in the present study, where the rates in conservative and dialysis patients were 36.7% and 30.5%, respectively. The detection of polyomaviruses in dialysis patients reinforces the idea that the pre-transplantation viral status should be considered an important risk factor for reactivation in the post-transplantation period because all recipient patients are immunosuppressed to avoid rejection of the allograft, which favors viral reactivation. In the transplanted group, the prevalence of polyomavirus was 20%, higher than that found by some authors [29,30,35] but lower than the majority described in the literature [20,36,37,38,39,40,41,42].

The low prevalence detected in the present study may be due to the different methodologies used in the transmission of polyomavirus infection. Currently, it is suggested that qPCR has greater sensitivity and specificity in monitoring polyomavirus infection or reactivation in transplant patients [20].

There are studies that found an association between the use of ATG and the occurrence of BKV viruria. Suwelack et al. [43] found higher relative risk with the use of ATG versus without, as well as ATG versus IL-2R inhibitor, with an incidence of BKV nephropathy confirmed by renal biopsy. Similarly, Schenker et al. [44] reported that, in a single ATG dose of 1.5 mg/kg body weight in living-donor transplant recipients, there was a 92% patient survival, an 83% graft survival, and a prevalence of urine BKV of only 5% over a 52.6 ± 31.9-month follow-up.

In addition to the influence of the ATG dose, as observed in the study by Schenker et al. [44], Parajuli et al. [45] highlighted the antibody reactivity panel (ARP), which analyzes every kidney transplant candidate on a quarterly basis, known in clinical practice as the “seroteca”. According to Parajuli et al. [45], when the ARP was less than 80%, ATG increased the probability of infection (*p* = 0.0001), while with an ARP greater than 80%, the use of ATG was not associated with the onset of infection (*p* = 0.35). However, after multiple regression analysis, ATG induction was associated with a significantly higher risk of BKV infection for patients with ARP greater than 80% (*p* = 0.02). In contrast, Schachtner et al. [46], in a cohort study conducted from 2004 to 2012, showed that lymphocyte depletion induction (anti-CD3 monoclonal antibodies: OKT3, ATG, alemtuzumab) was related to BKV viruria in 19% of cases, and with the use of IL-2R inhibitor, viruria was positive in 81%. On the use of ATG, Namba et al. [47], in a retrospective study of 112 specimens of renal graft biopsy (gold standard for the diagnosis of BKV nephropathy), showed that 83% of patients who used lymphocyte depletors had BKV nephropathy, without statistical significance (*p* = 0.42).

Regarding the maintenance of immunosuppression, the present study showed an association between infection by polyomavirus and the use of azathioprine, corroborating the findings of Brennan et al. [48], who reported a lower incidence of BKV viruria in patients undergoing the tacrolimus + azathioprine regimen (27%) versus tacrolimus + mycophenolate (46%; *p* = 0.03). In contrast, Suwelack et al. [43] found no higher risk of BKV infection between the use of mycophenolate or azathioprine.

Although viremia was not observed in any of the groups of patients investigated in the present study, it is important to highlight that Demey et al. [49], in a systematic review of the literature, did not observe a correlation between the use of ATG and viremia by BKV. However, they described as risk factors for the occurrence of BKV viremia in varying degrees: (i) the tacrolimus regimen, (ii) deceased donor, (iii) male recipient, (iv) history of previous transplantation, (v) age at transplantation, (vi) ureteral stent use, (vii) delayed graft function, and (viii) episodes of acute rejection.

Strategies to reduce immunosuppression have shown benefits in reducing the incidence of BKV-associated nephropathy. Thus, it is important to establish guidelines for the screening of infection and the prevention and treatment of patients.

## 5. Conclusions

In the present study, we observed infection by JCV and BKV in patients with chronic kidney disease in the different staging stages and their relationship with the use of immunosuppressants. Considering the importance of reactivating the infection to patient survival and graft loss, it is necessary to develop guidelines for the inclusion of screening tests for these viruses in patients with CKD to monitor the infection and define an appropriate therapeutic strategy, especially in Brazil, where polyomavirus screening is not part of the diagnostic workflow for individuals who are candidate kidney transplant donors or recipients.

## Figures and Tables

**Table 1:** Epidemiological characterization of the sex, age, and etiology of chronic kidney disease patients from the conservative, dialysis, and transplanted groups in Belém, Pará.

Characteristic	Conservative (*n* = 60)	Dialytic (*n* = 82)	Transplanted (*n* = 60)
Female	21 (35%)	33 (40.24%)	18 (30%)
Male	39 (65%)	49 (59.76%)	42 (70%)
Age in years (mean)	61.3	55.0	40.4
Etiology			
DM	13 (21.67%)	34 (41.46%)	4 (6.67%)
SAH	36 (60%)	24 (29.28%)	9 (15%)
Glomerulonephritis	5 (8.33%)	9 (10.97%)	11 (18.33%)
Lithiasis	20 (33.33%)	3 (3.66%)	2 (3.33%)
Polycystic kidneys	1 (1.67%)	3 (3.66%)	4 (6.67%)
Indeterminate	1 (1.67%)	3 (3.66%)	23 (38.33%)
Repeated UTIs	5 (8.33%)	2 (2.44%)	1 (1.67%)
Other	19 (31.66%)	4 (4.88%)	6 (10%)

**Table 2:** Prevalence of polyomavirus in the chronic kidney population by conservative, dialysis, and transplanted groups compared to the control group in Belém, Pará, Brazil, 2016.

PCR VP1	Conservative		Dialytic		Transplanted		Control		*p*
	*n*	%	*n*	%	*n*	%	*n*	%	
Positive	22	36.7	25	30.5	12	20.0	41	46.6	
Negative	38	63.3	57	69.5	48	80.0	47	53.4	0.0074
TOTAL	60	100	82	100	60	100	88	100	

**Table 3:** Prevalence of polyomavirus in the group with CKD as a function of glomerular filtration rate in Belém, Pará, Brazil, 2016.

VP1	Stage of CKD in the Conservative Group by GFR (mL/min/1.73 m^2^)										*p*
	1 (>90)		2 (60–89)		3 (30–59)		4 (15–29)		5 (<15)		
	*n*	%	*n*	%	*n*	%	*n*	%	*n*	%	
Positive	1	20.0	9	40.9	9	36.0	2	33.3	1	50.0	0.91
Negative	4	80.0	13	59.1	16	64.0	4	66.7	1	50.0	
TOTAL	5	100	22	100	25	100	6	100	2	100	

**Table 4:** Prevalence of polyomavirus in transplanted patients according to the type of immunosuppression therapy, at Ophir Loyola Hospital, Belém, Pará, Brazil, 2016.

VP1					
Therapy	Positive		Negative		*p*-Value
	*n*	%	*n*	%	
Induction Therapy					0.063
ATG	10	28.6	25	71.4	
IL-2R inhibitor	01	6.6	14	93.3	
Maintenance Therapy					0.015
Mycophenolate	04	11.4	31	88.6	
Azathioprine	08	40.0	12	60.0	
